# Identification and Characterization of the *DOF* Gene Family in *Phoebe bournei* and Its Role in Abiotic Stress—Drought, Heat and Light Stress

**DOI:** 10.3390/ijms252011147

**Published:** 2024-10-17

**Authors:** Kehui Zheng, Mengmeng Lv, Jiaying Qian, Yiran Lian, Ronglin Liu, Shuhao Huo, Obaid Ur Rehman, Qinmin Lin, Zhongyang Zhou, Xiaomin Liu, Shijiang Cao

**Affiliations:** 1 College of Computer and Information Sciences, Fujian Agriculture and Forestry University, Fuzhou 350002, China; zhkehui@fafu.edu.cn; 2 College of Forestry, Fujian Agriculture and Forestry University, Fuzhou 350002, China; youyosihq@163.com (M.L.); jiayingq0104@163.com (J.Q.); d1315068727@163.com (R.L.); 3 University Key Laboratory of Forest Stress Physiology, Ecology and Molecular Biology of Fujian Province, Fuzhou 350002, China; 4 College of Life Sciences, Fujian Agriculture and Forestry University, Fuzhou 350002, China; 18358678977@163.com (Y.L.); 18760030926@163.com (Q.L.); 5 School of Food and Biological Engineering, Jiangsu University, Zhenjiang 212013, China; huo@ujs.edu.cn (S.H.); obaid.sheikh@hotmail.com (O.U.R.); 6 State Key Laboratory of Tree Genetics and Breeding, College of Biological Sciences and Technology, Beijing Forestry University, Beijing 100083, China; yang18553738659@163.com

**Keywords:** *Phoebe bournei*, *Dof* gene family, polygenic, abiotic stress

## Abstract

*Phoebe bournei* is a second-class endangered and protected species unique to China, and it holds significant ecological and economic value. DNA binding one zinc finger (Dof) transcription factors are plant-specific regulators. Numerous studies have demonstrated that *Dof* genes are involved in plant growth, development and responses to abiotic stress. In this study, we identified and analyzed 34 *PbDof* gene members at the whole-genome level. The results indicated that the 34 *PbDof* genes were unevenly distributed across 12 chromosomes. We utilized the *Dof* genes from *Arabidopsis thaliana* and *P. bournei* to construct a phylogenetic tree and categorized these genes into eight subgroups. In the collinearity analysis, there were 16 homologous gene pairs between *AtDof* and *PbDof* and nine homologous gene pairs between *ZmDof* and *PbDof*. We conducted a *cis*-acting element analysis and found that *cis*-acting elements involved in light response were the most abundant in *PbDof* genes. Through SSR site prediction, we analyzed that the evolution level of *Dof* genes is low. Additionally, we assessed the expression profiles of eight *PbDof* genes under high temperature, drought, and light stress using qRT-PCR. In particular, *PbDof08* and *PbDof16* are significantly upregulated under the three stresses. This study provides foundational information for *PbDof* genes and offers new insights for further research on the mechanism of Dof transcription factors responding to stress, as well as the adaptation of *P. bournei* to environmental changes.

## 1. Introduction 

With the intensification of climate change, rising global temperatures and increased frequency of extreme weather events, plants are confronted with many challenges, including drought, high temperature, high salinity and strong light [1]. These stresses have a serious impact on plant growth, development and survival. To adapt to drastic environmental change, plants have developed a series of defense mechanisms to resist various kinds of adverse conditions. Transcription factors play a crucial role in plant responses to stress. They regulate gene expression by binding to *cis*-acting elements upstream of genes, thereby affecting the plant’s adaptation to stress [2].

DNA binding with one finger (Dof) constitutes a plant-specific transcription factor family. Belonging to the C_2_C_2_ single zinc lipoprotein superfamily, it is characterized by a particular conserved single zinc finger domain rich with Cys residues, and is thus called the Dof domain [3]. Dof proteins typically consist of 200 to 400 amino acid residues. They contain one single copy of the conserved Dof domain, as well as two additional domains: a highly conserved N-terminal DNA domain and a C-terminal transcriptional regulatory domain [4]. The binding domain is a highly conserved Dof domain consisting of 52 amino acids. This domain features a single zinc finger structure characterized by the motif CX_2_CX_21_CX_2_ [3]. Identified as a bifunctional domain, the Dof domain mediates DNA binding and plays a vital role in protein-protein interactions. [5]. Four conserved Cys residues in the single zinc finger structure are covalently bound to one Zn^2+^ and one change will lead to the loss of Dof protein activity [6] and the DNA-binding domain can interact with other proteins, highlighting its crucial role in protein-protein interactions [7]. Due to the highly conservation of the DNA-binding domain sequence in Dof proteins, they exhibit similar DNA-binding characteristics. The C-terminus has a transcriptional regulatory domain with multiple functions. This domain can interact with various regulatory proteins and activate gene expression [8]. The amino acids of the domain are poor conserved, and there is a great difference among Dof members, which leads to the diversity of Dof protein functions. The N-terminal and C-terminal regions of Dof can interact with a variety of regulatory proteins or blocking signals to activate and inhibit downstream regulatory factors [9].

**Figure 1:**
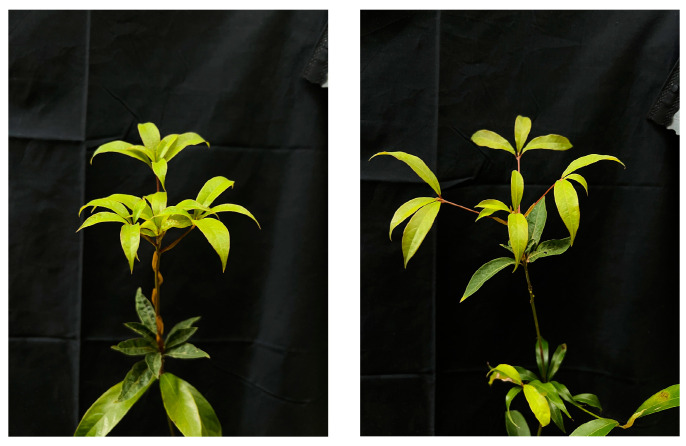
Image of the one-year-old *P. bournei* seedlings.

*Phoebe bournei*, an evergreen tree in the *Lauraceae* family, is an endemic species native to China [10] (Figure 1). It is suitable for growing in the warm and humid climate of the central subtropics and is mainly distributed in southern China. Although *P. bournei* is relatively widespread in southern China, most individuals are found in a sporadic and scattered distribution. This can be attributed to several factors. Firstly, the seeds have natural dormancy, which hampers germination and natural regeneration. Secondly, the tree has a slow growth rate in its natural habitat. Thirdly, it has suffered serious damage from human activities. As a result, the number of existing wild individuals is low and natural resources are becoming depleted. Therefore, it is classified as a nationally second-class rare species [11]. *P. bournei* has high economic, ecological, and ornamental value. *P. bournei* features a straight, well-formed trunk with dense and compact wood. Its durable aroma, attractive texture and ease of processing make it highly valuable for high-quality architecture, fine furniture, intricate carving, and shipbuilding. It is considered a precious timber species in China [12]. In addition, *P. bournei* is also widely utilized in fragrance extraction, cosmetics and other research and development fields [13].

To address the challenges posed by extreme climates and adapt to deteriorating environmental conditions, enhancing plant stress resistance has become an urgent priority. Current research indicated that Dof proteins are involved in various processes, including plant growth and development, hormone metabolism, cell cycle regulation and signal transduction [3,14,15,16,17]. Particularly, they play a key role in plant osmotic stress responses [18]. For example, the expression of most *BraDof* genes in Chinese cabbage is increased in response to high temperatures and drought stress. For wheat, the expression of *TaDof14* and *TaDof15* is upregulated under drought stress [18]. In poplar, the expression of *PtrDof14*, *PtrDof16*, *PtrDof25*, *PtrDof27*, *PtrDof37* and *PtrDof39* are continuously raised in both leaves and roots under ABA and osmotic stress [19]. In apples, *MdDof6* and *MdDof26* not only exert significant roles in abiotic stress but also involved in numerous molecular functions [20].

Since its initial identification in maize, Dof proteins have been identified and analyzed in numerous species. This indicates the widespread occurrence and potential significance of Dof proteins in different organisms [21]. There is significant variation in the number of *Dof* genes across different species. For example, only one *Dof* gene has been identified in *Chlamydomonas reinhardtii* [22], whereas there are 23 *Dof* genes in *Physcomitrella patens* [23]. Among higher plants, 36 Dof proteins have been detected in *Arabidopsis thaliana* [24], 31 in *Triticum aestivum* [25], 35 in *potato* [26], 30 in *rice* (*Oryza sativa L.*) [27], and 41 in *Poplar* (*Populus trichocarpa*) [19]. Additionally, there are 53 *Dof* genes in *olive* (*Olea europaea*) [28] and 20 in *Chrysanthemum morifolium* [29]. Nevertheless, there are comparatively few studies on Dof proteins in *P. bournei*. While the *Dof* gene family has been well-studied in model species, there is still a lack of information in non-model species. Especially in trees of economic and ecological importance like *P. bournei*. Research on the biological information and expression patterns of its gene family is extremely limited. Our study provides the first comprehensive analysis of the *Dof* gene family in *P. bournei*, filling a critical gap in the current knowledge. This study conducts the comprehensive determination and analysis of the *Dof* gene family in *P. bournei* at the whole-genome scale, encompassing its phylogenetic relationship, gene structure, physicochemical properties of proteins, *cis*-acting elements, collinearity and syntenic analysis, Ka (non-synonymous substitutions rate), Ks (synonymous substitutions rate) and Ka/Ks (omega(ω)) value, tissues expression profiles, SSR locus prediction and real-time quantitative PCR expression level under abiotic stress. Additionally, we investigated the variation in expression levels of the *PbDof* genes under abiotic stress. The experimental methodology of the relative expression of *Dof* gene in *P. bournei* under drought, heat and light stress was derived from the published paper in our laboratory. It has been revealed that this experimental method has an important reference for revealing the key physiological information of *P. bournei* [30] This offers a reference for future studies on the functions of Dof transcription factors and laying a foundation for the study on the stress resistance of *P. bournei*.

## 2. Results

### 2.1. Phylogenetic and Evolutionary Analysis of PbDof Proteins in P. bournei

In the genome of *P. bournei*, we identified 34 *Dof* genes, which were designated *PbDof01* through *PbDof34* (Table 1). Analysis using Expasy indicated that the protein length of the *PbDof* genes ranged from 122 to 1191 amino acids (aa), with an average length of 214.85aa. Moreover, only *PbDof19* protein had an instability index value lower than 40, which was a critical stable protein, while all other proteins were unstable [31]. The average aliphatic index (AI) of PbDof proteins was 54.04, indicating a low abundance and relative content of non-polar amino acids, which suggests poor thermal stability of the proteins. There were negative Grand Average of Hydropathicity (GRAVY) data for all the PbDof protein sequences, indicating that PbDof proteins had hydrophilic properties. The isoelectric point (PI) of PbDof proteins averaged 7.94, with a range from 5.28 (*PbDof12*) to 9.59 (*PbDof19*), showing that these proteins contain more basic amino acids. The average molecular weight (MW) of the proteins is 34,349 kDa. Subcellular localization prediction indicated that, with the exceptions of *PbDof02*, *PbDof12*, *PbDof19*, and *PbDof34*, all other *PbDof* proteins are located in the nucleus.

### 2.2. Analysis of the Structure and Motif of PbDof Genes

**Figure 2:**
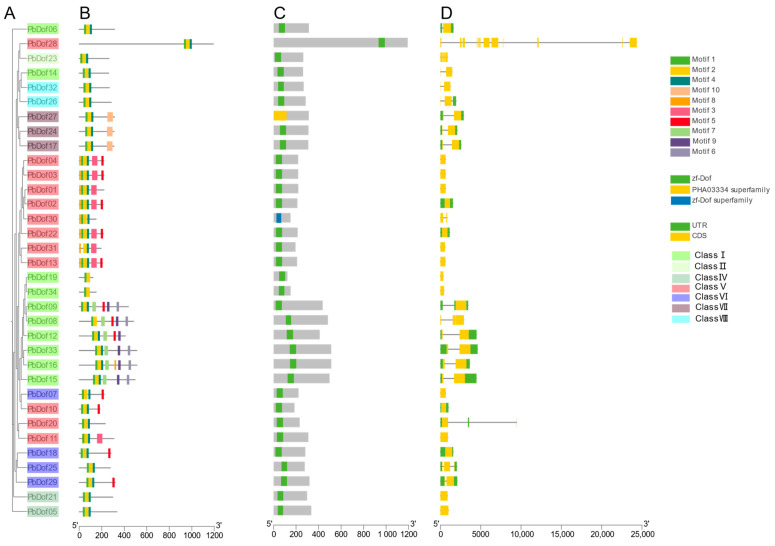
Comprehensive analysis of PbDof genes in *Phoebe bournei*. (**A**) The phylogenetic trees developed to elucidate the relationships among PbDof members, highlighting evolutionary patterns and potential functional similarities or divergences. (**B**) Conserved motifs in PbDofs, illustrating their distribution and prevalence across different proteins. (**C**) Analysis of the conserved domains within PbDofs, emphasizing the structural elements essential for their transcription factor activity and interaction with other proteins. (**D**) Gene structures of the *PbDof* genes, representing the exon-intron organization of the *PbDof* genes, with particular focus on variations among different subfamilies.

In this study, the MEME website was used to predict the conserved motifs in PbDof proteins. Furthermore, we examined the relationship between the structural diversity and evolution of *P. bournei* proteins by identifying 10 conserved motifs (Figure 2A,B). The results indicated that Dof motifs were present in all 34 PbDof proteins, confirming their membership in the Dof family. The type and number of motifs were similar across different subfamilies, with motif2 being present in all PbDof proteins. However, there were some intra-groups specific motif2 that may be the PbDof protein that evolved different structures to perform different functions. For instance, in group VII, there existed the specific motif 10; motif 9 and motif 6 were only present in group I; motif 8 exists only in group V. Within the same group, the number motifs were varied from 2 to 7. Subfamily I contains the highest number of motif proteins among PbDof proteins, suggesting that genes in this subfamily have undergone a longer evolutionary period. Moreover, we identified two conserved domains in *PbDof* genes: zf-Dof and PHA03334. (Figure 2C).

Analysis of exon/intron structure and exon count revealed that subfamilies V contains more introns than other subfamilies, classifying it as intron-rich (Figure 2D). Interestingly, *PbDof28* has the longest intron, measuring 20,790 bp. It is speculated that the genes of the subfamily Class V may have higher evolutionary potential and are involved in many regulatory functions. Seventeen *PbDof* genes lacked untranslated regions (UTRs). The analysis indicates that highly homologous PbDofs share similar motif compositions, conserved domains and gene structures. Variations in the evolutionary levels of PbDofs across different subfamilies suggest that these proteins may have similar functions.

### 2.3. Protein Evolution and Collinearity Analysis of PbDof Gene

**Figure 3:**
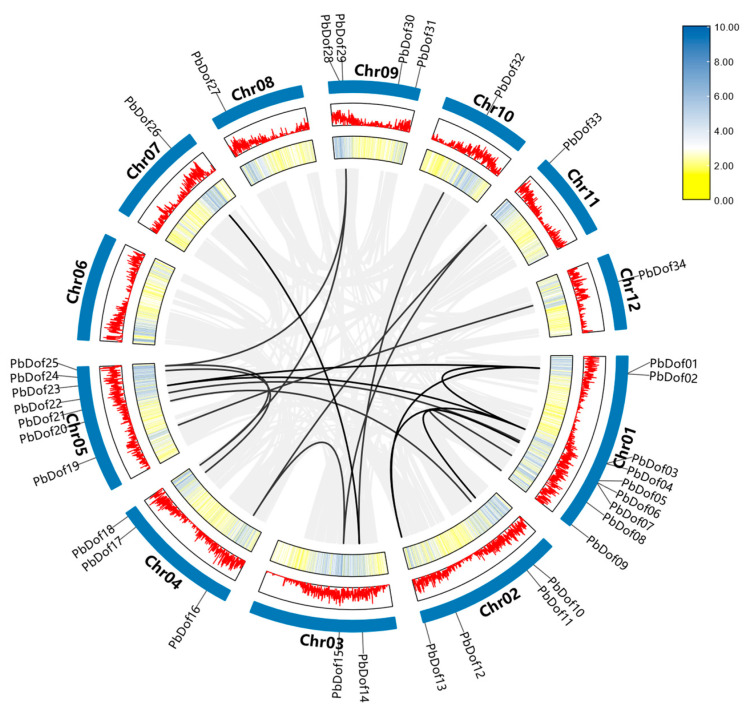
A circular presentation of the genomic map of *Phoebe bournei*. The outer segments of the circle represent the 12 assembled chromosomes. Each segment is labeled from chromosome 1 (Chr01) through chromosome 12 (Chr12). Moving inward from the outermost part of each chromosomal segment, the first circle indicates the nucleotide positions in megabases (Mb), providing a scale for genetic mapping. Adjacent to this, the gene density is visualized, with peaks indicating areas of higher gene concentration. The gray line in the innermost circle represents all replications in the *P. bournei* genome gene pairs, the black line indicates the *PbDofs* collinear gene pair.

We further analyzed the localization of *PbDof* genes on the *P. bournei* chromosomes (Figure 3). The majority of *PbDof* genes were unevenly distributed across 12 chromosomes, excluding chromosome 6. Notably, chromosome 1 contained the highest number of *PbDof* genes, with nine genes identified. The second most prominent was chromosome 5, which had a total of seven *PbDof* genes. Each of the other 9 chromosomes distributes 1–3 small *PbDof* gene members and there was no *PbDof* gene on chromosome 6. More than half of the chromosomes have the *PbDof* genes from subfamily, which are distributed across the majority of the chromosomes.

From an evolutionary perspective, previous studies indicate that tandem duplication events commonly occur within specific gene families and are closely associated with the gene amplification, which is also related to abiotic stresses [32]. Tandem duplication events are defined as more than two repeated genes within a region less than 200 kb [33]. Intraspecific collinearity showed that 34 *PbDof* genes formed 24 pairs of collinear gene pairs. Only four *PbDof* genes lacked corresponding collinear gene pairs, and the tandem duplication events were more frequent on chromosomes 1 and 5. Given that frequent tandem duplication events aid plants in adapting to environmental stress [34], it can be inferred that the *PbDof* family in *P. bournei* has a strong response to abiotic stress.

### 2.4. Phylogenetic Analysis of PbDofs

**Figure 4:**
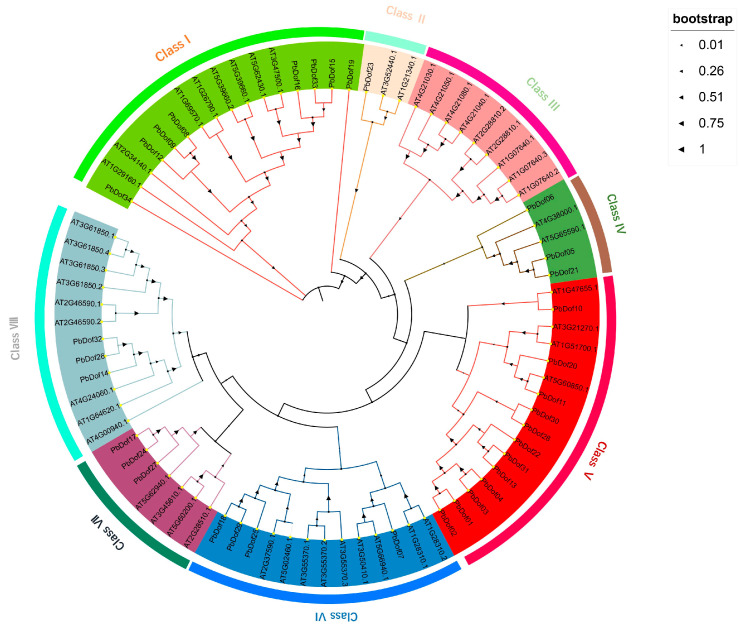
Phylogenetic tree of *Dof* gene family in two species. Different branch colors and background colors represent eight groups and black triangles on branches show bootstrap values.

To classify the PbDof family and emphasize the differences between *P. bournei* and other plants, 34 hypothetical *PbDofs* and 47 reference *AtDofs* (Plant TFDB) were selected to construct the phylogenetic tree (Figure 4). According to the previous classifying of *A. thaliana* [3], the phylogenetic tree was divided into eight subclasses: class I contained eight *PbDofs* and eight *AtDofs*; class II included three members, comprising one *PbDof* and two *AtDofs*; class III consisted of nine Dof proteins from *A. thaliana*, with no corresponding counterparts in *P. bournei*. Class IV, with five members, included three *PbDofs* and four *AtDofs*. Class V consisted of 12 *PbDofs* and 4 *AtDofs*, while Class VI had 4 *PbDofs* and 9 *AtDofs*. In Class VII, there were three *PbDofs* and four *AtDofs*, and the final subclass, Class VIII, contained three *PbDofs* and nine *AtDofs*. Among the eight classes, Class V had the highest number of members (12 Nos), whereas Class II had the fewest (1 Nos). Moreover, the results showed that the homology among *PbDofs* was generally higher than the homology between *PbDofs* and *AtDofs*.

In subfamily V, *PbDof11* showed a high homology with *AT5G60850.1* (*OBP4*), suggesting that *PbDof11* may also play a similar role in hormone signaling [35] and callus formation [36]. Similarly, *PbDof07* and *AT3G50410.1* (*OBP1*) were in the same clade, so it could be speculated that *PbDof07* may also play a vital role as a transcriptional regulator in regulating cell cycle during embryogenesis, sprout and lateral roots formation [16].

### 2.5. Ka, Ks and Ka/Ks of PbDofs

**Figure 5:**
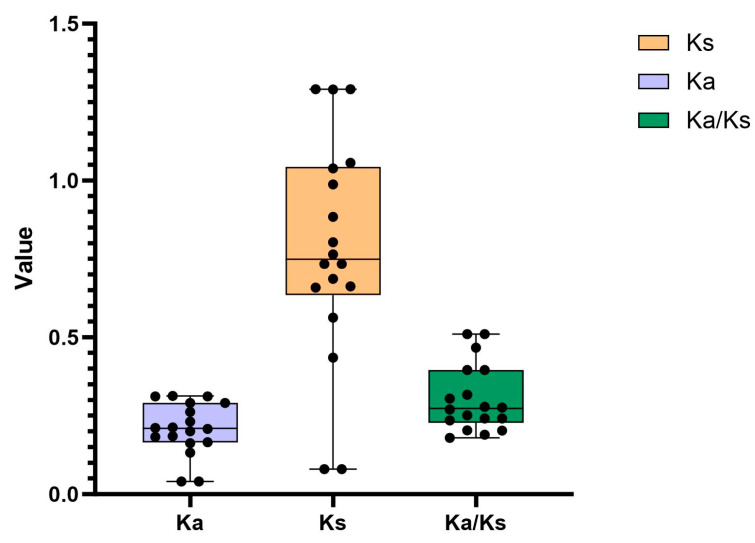
The Ka, Ks and Ka/Ks value of the collinear *PbDofs* gene pair.

We calculated the amount of non-synonymous substitution sites (Ka) for 19 pairs of *PbDof* homologous genes to analyze the intensity of natural selection (Figure 5). Ka/Ks ratios was also calculated, with the specific Ka/Ks values shown in the Supplementary Tables. Ka/Ks > 1 represents positive selection effect. On the contrary, Ka/Ks < 1 means to have a depuration selection effect. Neutral selection can only be identified with the result of Ka/Ks = 1. As a result, 18 pairs of *PbDofs* had low Ka/Ks values, with only one homologous gene pair exhibiting a Ka/Ks ratio exceeding 0.5. The Ka/Ks values varied from 0.179 (*PbDof15*/*PbDof16*) to 0.510(*PbDof08*/*PbDof09*), suggesting that members of the *PbDof* gene family have experienced intense purification selection, effectively removing a significant number of deleterious mutations throughout their evolutionary history. The Ks values of the collinearity gene pair *PbDof17*/*PbDof24*, *PbDof18*/*PbDof25*, and *PbDof18*/*PbDof29* could not be calculated and it is speculated that many of the sites that can have homologous mutations in these collinearity genes have been synonymously replaced, resulting in large sequence divergence at the nucleic acid level.

### 2.6. Syntenic Analysis of PbDofs Genes

**Figure 6:**
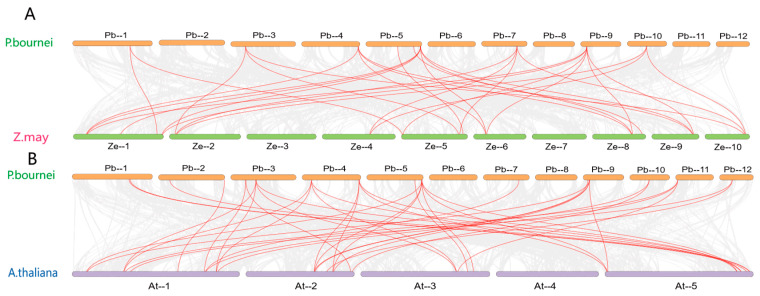
(**A**) The collinearity analysis between *Dof* genes of *P. bournei* and *A. thaliana*. (**B**) The *Dof* genes collinearity analysis between *P. bournei* and *Z.mays*. Different species names and chromosomes are represented by different colors. The red line indicates the homologous *Dof* gene pairs between other species and *PbDofs*, the grey line indicates all homologous gene pairs on the chromosome and the number in parentheses after the species name indicates the number of collinearity pairs between the *Dof* gene of the species and *PbDof* genes.

To assess the collinearity of the *PbDof* gens with species across several major evolutionary branches of angiosperms, we performed collinearity analyses using a core dicot (*A. thaliana*) and a monocot (*Z. mays*) with *P. bournei* and we identified 47 and 52 genes in *A. thaliana* and maize, respectively. A total of 16 and 9 homologous gene pairs were identified between 19 *AtDof* genes and 15 *ZmDof* genes, respectively. (Figure 6A,B). Detailed statistical data are provided in the Supplementary Tables. Based on the collinearity of *PbDof* genes, 34 genes on chromosomes were classified, and there were 7 colinear genes with basal dicots and monocot (*PbDof14*, *PbDof18*, *PbDof25*, *PbDof23*, *PbDof26*, *PbDof29*, *PbDof32*). By analyzing the collinearity between *P. bournei* and *A. thaliana*, as well as between *P. bournei* and *Z. mays*, tandem repeat genes conserved across these three species can be identified. Studying these conserved tandem repeat genes provides valuable insights into the diversity of gene families.

### 2.7. Analysis of Cis-Regulatory Elements of Dof Family Genes in P. bournei 

**Figure 7:**
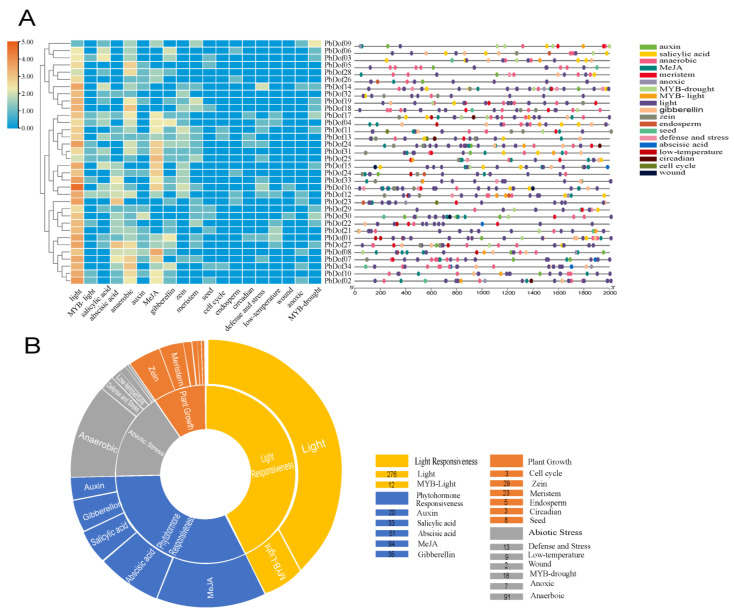
Predicted *cis*-element on the promoter of the *PbDof* genes. (**A**) The abridged general view of distribution of *cis*-element positions in each *PbDof*. (**B**) The pie chart shows the number of cis-acting elements in *PbDofs*. Different colors indicate different core *cis*-acting elements in response.

We have submitted 2000 bp upstream of the *PbDofs* from the initiation site to the PlantCARE database. Figure 7A,B indicates that the functions of *cis*-elements can be divided into plant hormone-responsive elements, abiotic stress elements, light-responsive elements and plant-growth elements. Each element has 5, 6, 1 and 7 isoforms, respectively.

The form and amounts of *cis*-acting elements in the promoter of *PbDofs* were counted. Altogether 781 *cis*-acting elements were identified (Table S1). There were 338 light responsive elements in most of the promoter regions, followed by 242 plant hormone responsive elements, 126 abiotic stress-responsive elements, and 75 plant growth elements. We can also find that except for the core *cis*-elements, CGTA-motif and TGACG-motif (methyl jasmonate), ABRE motif, GA-responsive, auxin-responsive (AuxRE, TGA element) in hormone-response type. Types of response to abiotic stresses include anaerobic-inducible (ARE elements), light-response (G-box, TCT-motif elements) and low temperature responses (LTR elements), also as regulatory motifs associated with tissue-specific expression (e.g., endosperm and palisade tissues), development or cell differentiation (e.g., circadian). Result displayed that light-responsive element was not only had plenty amounts of *cis*-elements but also broadly existed in the *PbDof* promoter region. In summary, the consequence showed the functional expression of *PbDof* gene was regulated by *cis*-acting elements related to light, plant hormones, abiotic stresses and plant development.

### 2.8. The Expression Patterns of the Dof Genes in P. bournei

**Figure 8:**
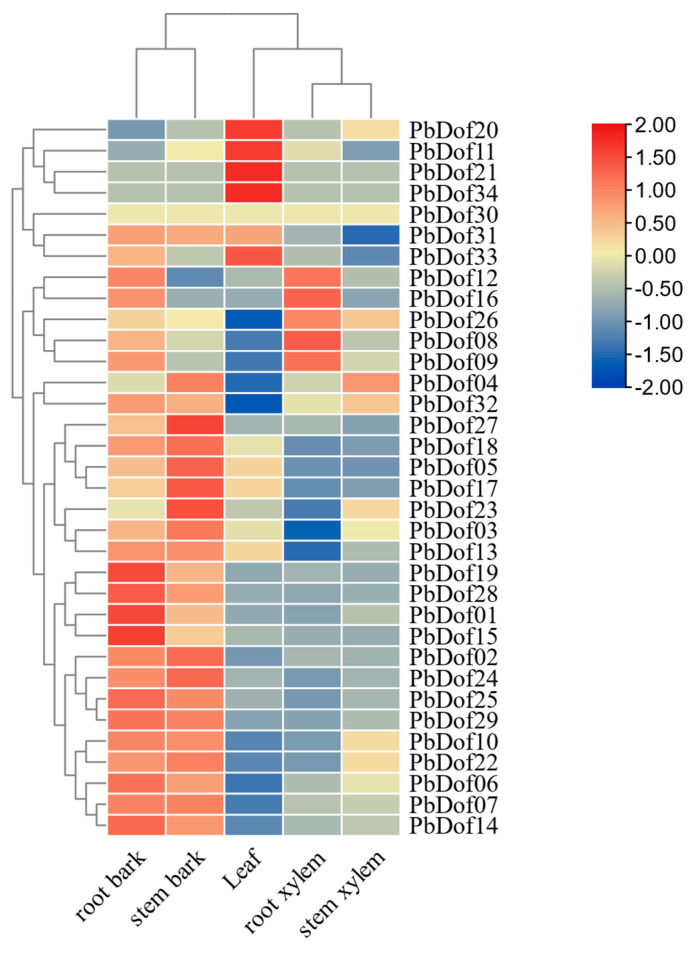
Expression profiles of 34 *PbDof* genes in different tissues. Gene expression patterns in five tissues, including root bark, stem bark, leaf, root xylem and stem xylem. Schematic diagram shows expression level: red color lump represents high expression quantity; blue color lump means a low expression quantity.

Based on the Fragments Per Kilobase of transcript per Million mapped reads (FPKM), we used the program R to construct the heat map of the *PbDofs*. Then, we analyze the discrepancy of expression patterns of *PbDofs* by including five different plant tissues and collecting their gene expression data (Figure 8). These five plant tissues include root bark, root xylem, leaf, stem bark and stem xylem. The result suggests that in leaf, the *PbDof20*, *PbDof11*, *PbDof21* and *PbDof34* had the highest expression and *PbDof26*, *PbDof08*, *PbDof09*, *PbDof04* and *PbDof32* had the lowest expression. The *PbDof19*, *PbDof28*, *PbDof01* and *PbDof15* had a similarly high expression in root bark, with moderate expression in other tissues. Moreover, the *PbDof27* was highly expressed in stem bark. Furthermore, *PbDof16* and *PbDof08* had the highest expression in root xylem. On the contrary, *PbDof16* and *PbDof08* had lower expression in the stem xylem.

### 2.9. SSR Locus Prediction of PbDofs

SSR markers are considered to be neutral molecular markers randomly distributed in the genome and SSR length is characterized by high variability and polymorphism. SSR is widely used in gene mapping, genetic diversity analysis and phylogenetic studies. To preliminarily understand the gene expression and distribution of *P. bournei*, candidate genes were screened from the massive transcript information obtained, SSR sites were mined and polymorphism detection was carried out. The MISA website was chosen to seek SSR sites in *PbDof* gene sequences using the following parameters: a minimum number of replicates of 2–5 baes, 10, 5, 4, 3, 3, 3 and a distance of less than 100 bps between the two SSRs was combined into a composite SSR. In summary, 18 SSR loci were found by searching the Unigene of 34 *Dof* candidate genes (Table 2). SSR repeat types vary greatly in proportion to the number of trinucleotides. Among them, trinucleotides accounted for 77.8%, which was the most abundant and the dominant repeat type. The proportion of pentanucleotides and hexanucleotides are only 5.6% and 11.1%. SSR characteristic analysis offers a theoretical foundation for screening and developing SSR molecular markers for the following study. It is proposed that low-level repeat units indicate that the species has a high level of evolution, while species with a large proportion of high-level repeat units have a low evolutionary time or mutation frequency [37]. Therefore, it can be conjectured that the *Dof* gene family has a low level of evolution.

### 2.10. Expression Analysis of Dof Family Genes in P. bournei Under Drought, Heat and Light Treatments

Drought, heat, and light stresses can affect plant growth, and these abiotic stresses typically occur at the transcriptional level. We investigated the role of *PbDof* genes in *P. bournei* response to drought, heat, and light stress by selecting seven *PbDofs* from seven subfamilies (I–VII) using quantitative real-time polymerase chain reaction. The treatment process included polyethylene glycol (PEG), heat and light treatment (Table S2).

When plants are under drought stress, the expression patterns of all *PbDof* could be known as ‘first rising and the falling’, *PbDof22*, *PbDof16*, *PbDof03*, *PbDof33* and *PbDof15* arrived at the peak expression level at 4 h and then declined, while *PbDof08* and *PbDof09* spent 8 h to determine to the highest expression level and decrease afterward. The maximum expression levels of *PbDof08* and *PbDof09* were approximately 15-fold higher than the 0 h, and the highest expression quantity of *PbDof08* and *PbDof09* were approximately 8-fold higher than the expression quantity of the control group, even after the 24 h drought stress time. After two-way analysis of variance, it was found that drought and heat stress both had a huge impact on the expression of *PbDof* genes, but the expression of *PbDof* under heat stress was more obvious than that under drought stress (*p* < 0.05). The majority of the genes were expressed at the same time and decreased at the same time. All seven *PbDof* genes reached the highest expression level at 12 h. The response of *PbDof08* to drought stress was very significant, reaching approximately 50-fold the expression level of the control group at 12 h. The expression of *PbDof* under light treatment was more regular than that under heat and drought stress and the expression quantity of *PbDof* gene showed an expression pattern increasing with time. All seven *PbDof* genes reached their maximum expression of *PbDof16* was significant, reaching approximately 40-fold higher than the expressed quantity at 0 h.

**Figure 9:**
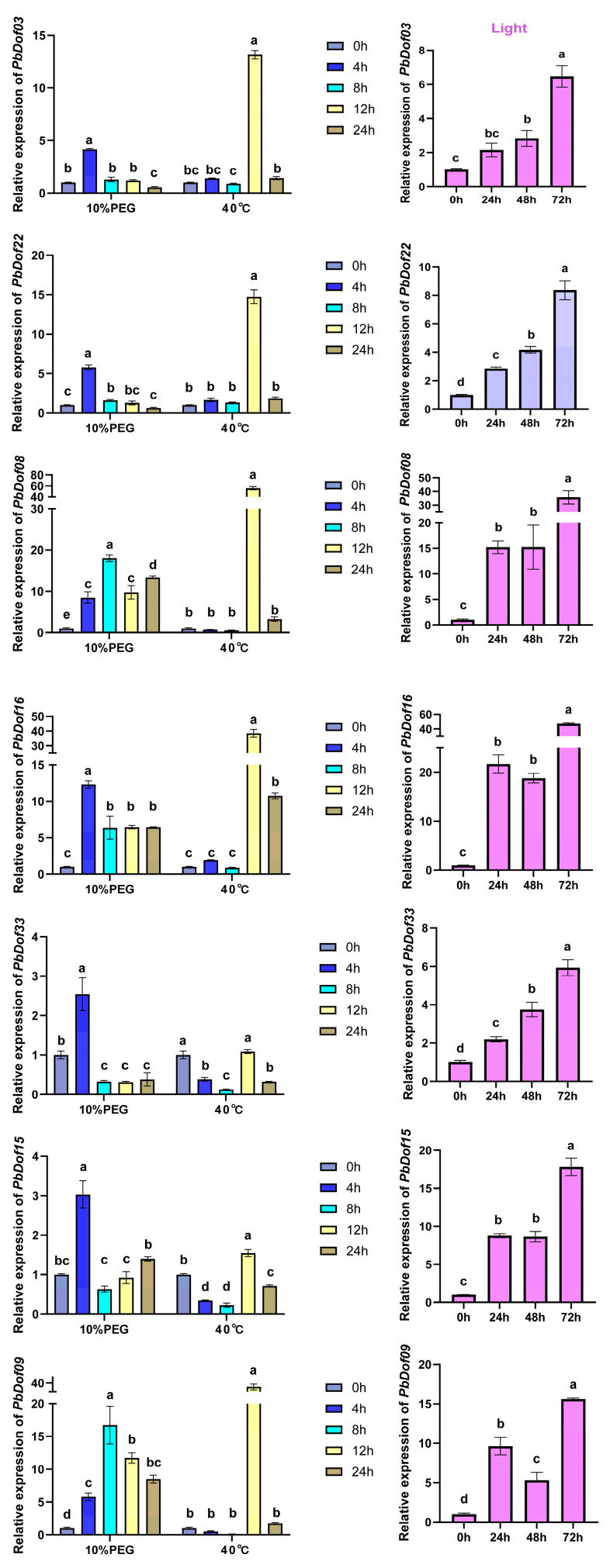
Real-time quantitative PCR (RT-qPCR) expression levels of selected *PbDof* genes under drought (10%PEG) heat (40 °C) and light (3000 lx) treatment. *PbDof* gene expression levels were analyzed by RT-qPCR. The sample size of the experiment is 3. The purple color represent light treatment, and the other colors represent the treating time. Significant differences (*p* < 0.05) determined by the LSD test, expressed by different letters above the bar.

In conclusion, all of the gene expression levels reached the highest peak during a certain period under drought, heat and light treatment. The expression of *PbDof* genes showed an upward trend in all treatment periods, which suggested that *PbDof* may be crucial to plants coping with three stresses. Both *PbDof08* and *PbDof16* had significant responses under drought, heat, and light stress. The highest expression levels of *PbDof08* gene and *PbDof16* gene under heat stress were significantly higher than those under drought stress (*p* < 0.05). It was speculated that the *PbDof* gene in *P. bournei* was dominated by a few specific *PbDof* genes in drought, heat and light treatment (Figure 9).

## 3. Discussion

*P. bournei*, an endemic and endangered species in China, thrives primarily in the subtropical monsoon regions. Known for its tough, tensile wood that is both workable and aesthetically pleasing, *P. bournei* hold significant economic value. However, the survival and flourishing of *P. bournei* are under threat from global warming, which is expected to intensify heat and drought conditions in its natural habitat [38]. Such environmental stresses are predicted to increasingly disrupt the normal growth, timber yield, and quality of *P. bournei* wood. Given the rapid climatic shifts and the changes in the habitat of *P. bournei*, understanding its mechanisms of stress resistance becomes crucial. Investigating how *P. bournei* adapts to repeated drought, heat and excessive light stress could offer insights critical for conserving and perhaps enhancing the resilience of this valuable species.

The Dof (DNA binding with one finger) proteins is a part of a family of plant-specific transcription factor (TF) genes. A large number of studies have shown that it can be widely involved in plant development and responses to abiotic stress. Previous studies have found the Dof proteins in economic crops such as *A. thaliana*, *Gossypium hirsutum*, *Brassica napus*, tomato and annual Alfalfa *(Medicago polymorpha)* [39,40,41,42,43]. So far, however, no studies have been confirmed on the *Dof* genes in *P. bournei*.

The Dof TFs possess Dof DNA-binding domains typically situated near the N-terminus. These highly conserved domains consist of 52 amino acid residues, including a chain of CX_2_CX_21_CX_2_C and a characteristic zinc finger configuration that binds zinc (Zn^2+^) and a downstream alkaline region [44]. These properties exhibit very short classical recognition sequences for Dof TFs. Several studies indicating that Dof proteins can work in combination with other transcription factors [9]. In this study, we compared the *AtDof* genes with the *P. bourinei* genome and identified 34 related *Dof* genes in *P. bournei*. Phylogenetic analysis of *A. thaliana* and *P. bournei* revealed their evolutionary relationship (Figure 6A). The *PbDof* genes identified in the genome of *P. bournei* are unevenly distributed on chromosomes, which may be due to unequal gene duplication of chromosomal segments. No *PbDof* gene was found on Chr06, possibly due to fragment loss or chromosomal translocation during evolution. Among these chromosomes, chromosome 1 contains the largest number of *Dof* genes and it can be speculated that this chromosome is the key chromosome to respond to abiotic stress (Figure 3). An evolutionary tree was built using the Dof proteins of *A. thaliana* and *P. bournei*, and *PbDof* was divided into seven subfamilies (Figure 4). By examining the domains and conserved sequences of 34 *PbDof* genes, we noticed that all members of the *PbDof* gene family had similar motif composition, conserved domains, and gene structures (Figure 2). Among them, we found that there is the longest exon in *PbDof28*, which may indicate that the gene structure has more complex functions. Members of the subfamily I have the highest number of motifs, suggesting that they have undergone extended evolutionary changes. 

By predicting the *cis*-acting elements of *P. bournei* gene family, we can further understand the gene composition of *PbDofs* and study its function in plant growth, development, and environment adaptation (Figure 7). Various studies have shown that Dof TFs are among the most suitable candidates for improving plant stress resistance in molecular breeding [45,46,47,48]. In this research, we found various regulatory motifs, with photoreactive elements being the most prominent and widely distributed (Table S1). In *Arabidopsis*, *Dof* genes are involved in seed germination, with red light pulses acting as a signal to initiate the process [49]. Plant seedlings undergo photomorphogenesis after germination, and light mediates these developmental processes primarily through the photoreceptors: phytochrome A (phyA) and phytochrome B (phyB), through the control of downstream signaling molecules and hormone levels. Previous studies have demonstrated that *A. thaliana*’s Dof protein COG1(COGWHEEL 1) is involved in phyA and phyB-mediated seedling development processes [50]. The Dof protein OBP3 of *A. thaliana* regulates photosensitizing pigments and cryptochromes by participating in multiple light signaling pathways to affect the photomorphogenesis of seedlings [51]. In addition, the transcription factors DAG1 and DAG2 are involved in phyB-mediated signal transduction, which affects plant germination [49]. Overexpression of the OBP3 transcription factor causes *sob1-D* mutation, which restrains the development of the plant’s long hypocotyls [15]. In addition to light treatment, the *Dof* gene family in plants also showed strong resistance under other abiotic stress conditions. For example, the expression of Ib*Dof16* in sweet potato was upregulated under heat or drought stress [52]. SICDFs have been shown to respond to heat and drought stress, crucial for enhancing heat tolerance and drought tolerance [53].

With drought, high temperature, and light treatments, the expression patterns of seven *PbDof* genes were analyzed by RT-qPCR (Table S2). Notably, the expression of all genes was enhanced under three abiotic stresses, among which *PbDof08* and *PbDof16* were significantly expressed under light, heat and drought stresses. Under drought stress, the expression levels of each gene showed differences at different treatment times. The expressions of *PbDof03*, *PbDof22*, *PbDof16*, *PbDof33* and *PbDof15* were upregulated until 6 hours. *PbDof08* and *PbDof09* were upregulated under drought stress. All of the seven genes reached the peak of upregulation on the 12th hour of heat stress, and *PbDof16* and *PbDof33* genes were the most sensitive to heat treatment, which may play a key role in plant response. The expression of five genes, *PbDof03*, *PbDof22*, *PbDof08*, *PbDof33* and *PbDof15* was continuously upregulated within 72 h of light treatment. Moreover, *PbDof08* and *PbDof16* played a very significant role in light stress treatment. *PbDof08* and *PbDof16*, together with *COG/Dof1.5* and *CDF4/Dof2.3*, belong to the same subfamily II and are located in the same clade. Overexpression of *COG1/Dof1.5* and *CDF4/Dof2.3* attenuates various reactions to red and far-red light. These two genes are negative regulators of photosensitin-mediated photo-responses and are consistent with the *PbDof* transcriptome results [54,55]. Therefore, we speculated that *PbDof08* and *PbDof16* were the main photoregulatory genes in *P. bournei.* In conclusion, the *PbDofs* gene first increased and then declined under drought and heat. In light stress, it showed a gradual upward trend. We also demonstrate that *PbDof08* and *PbDof16* play a crucial role in heat and light tolerance in *P. bournei*, providing valuable insights into the stress resistance of this species.

**Figure 10:**
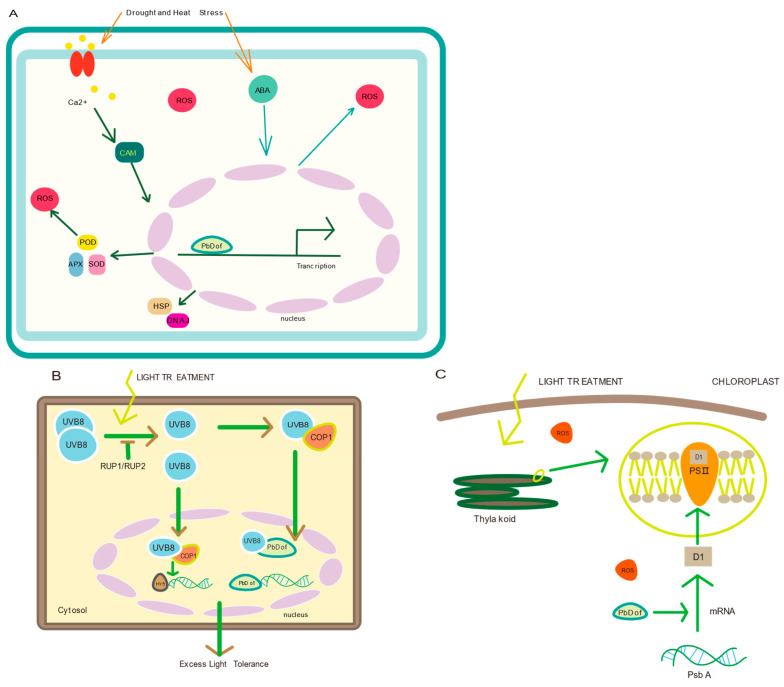
(**A**) Prediction of response mechanism of *Dofs* to drought and high temperature stress. The two orange arrows on the top stand for Drought and heat stress, respectively. Drought or heat stress promotes the gene expression of POD, APX and SOD enzymes to degrade the excess ROS in the cytoplasm. HSP: heat shock protein, SOD: superoxide dismutase, POD: peroxidase, APX: ascorbate peroxidase, and DHAJ: a member of the heat shock protein family. (**B**) Mechanism of prediction of *Dofs* response to light stress in plant chloroplasts. On the right shows a magnified image of the thylakoid bilayer membrane. The prediction of protein on the membrane is expressed. (**C**) Speculate on the mechanism of *Dofs* response to light stress in plant cells. Yellow arrow indicates that plant cell is under light stress. The UVB8 act as the signaling molecule in response to light stress.

When plants are exposed to abiotic stress, the levels of ROS [56], Ca^2+^ concentration and ABA in plant cells increase within plant cells. ABA is a key factor in regulating the plant response to abiotic stress. At present, only a few studies have shown that *Dof* gene regulates abiotic stress response through ABA-dependent signaling pathway to reduce intracellular ROS concentrations. However, the precise mechanisms underlying this process remains poorly understood. There is also an ABA-independent pathway that contributes to the plant’s response to abiotic stress. Under drought and heat stress, *Dof* gene expression promotes the production of genes encoding heat shock proteins (HSFs), peroxidases and DNAJ proteins, which work together to maintain ROS homeostasis in cells and participate in osmotic protection (Figure 10A) [57]. Plants possess two systems-photoreceptors and chloroplasts-that receive light information from the environment. Both systems have mechanisms to respond to excess light exposure. The UVB8 receptor receives UV-B light from excess light. A single UVB8 molecule can directly bind to transcription factors to regulate the light stress response [58]. After plants were subjected to light treatment, photosystemII (PSII) on the thylakoid membrane of chloroplasts becomes highly susceptible to photoinhibition [59]. The UVB8 molecule may bind to HY5 and PbDofs, respectively, and thus jointly regulate light stress (Figure 10B). In addition, excess ROS accumulates within the chloroplast matrix. The core subunit protein (D1 protein) of the photosystem II complex in the thylakoid membrane is particularly sensitive to ROS and is easily degraded by oxidative damage. At the same time, ROS also inhibits the translation of psb A, the gene encoding D1 protein [60]. The primary repair mechanism in plant chloroplasts for PSII following light stress involve ROS scavenging and the D1 repair cycle. ROS in the chloroplast matrix is degraded by ROS scavenging enzymes [61]. It is speculated that the Dof transcription factor was involved in regulating the expression efficiency of the *psb A* gene under light stress (Figure 10C).

## 4. Materials and Methods

### 4.1. Genome-Wide Identification of PbDof Proteins 

The complete genome sequence and annotation files of *P. bournei* were obtained from China National Gene Bank Data Base (https://db.cngb.org/search/project/CNP0002030/, accessed on 2 July 2024) [30]. We obtained the RNA-seq data of different tissues of *Phoebe bournei* from the NCBI database (https://www.ncbi.nlm.nih.gov/sra/?term=PRJNA628065, accessed on 2 July 2024). The DOF protein sequence of *A. thaliana* was retrieved from the TAIR (https://www.arabidopsis.org/, accessed on 2 July 2024). Taking the AtDof protein sequence as the query sequence, we conducted a homologous search against the *P. bournei* database via Blastp in TBtools-IIv2.10 software [30]. Simultaneously, the Hidden Markov Model (HMM) of the DOF protein (PF02701) was retrieved from the online website Interpro (https://www.ebi.ac.uk/interpro/, accessed on 2 July 2024). Then, the software TBtools-IIv2.10 was utilized to search the genomic protein database of *P. bournei*. After combining the sequences obtained by Blastp and HMM, the conserved domains were confirmed by Batch CD-search in NCBI (https://www.ncbi.nlm.nih.gov/Structure/bwrpsb/bwrpsb.cgi, accessed on 2 July 2024). Eventually, the members of the *P. bournei Dof* gene family were acquired (genome.jp). The physicochemical properties of members of the PbDof family, including the theoretical PI, molecular weight, and the number of amino acids were analyzed by the online website ExPASy (https://www.expasy.org/, accessed on 3 July 2024) and WOLF PSORT (https://wolfpsort.hgc.jp/, accessed on 3 July 2024). Finally, we predict the subcellular localization of the *PbDof* family members by using the online website WOLF PSORT (https://wolfpsort.hgc.jp/, accessed on 3 July 2024).

### 4.2. Evolution Analysis and Chromosome Localization

The whole-genome file and annotation file of *P. bournei* was used to perform visualization using TBtools-IIv2.10 software in order to determine the chromosomal location of the *PbDof* genes. The whole-genome annotation information of *A. thaliana* and rice were downloaded from the TAIR website and PlantTFDB (https://planttfdb.gao-lab.org/, accessed on 5 July 2024) website, respectively. The “Dual synteny Plot for MC scanX” in TBtools-IIv2.10 was used to analyze the collinearity relationship between the aforementioned species and *P. bournei*. The “Advanced Circos” function in TBtools-IIv2.10 software was used to visualize the *PbDof* gene duplication event, and calculate the Ka, Ks and Ka/Ks values.

### 4.3. Evolutionary Tree Analysis of PbDof Proteins

The evolutionary tree of the Dof family for *P. bournei* and *A. thaliana* was created by the maximum-likelihood (ML) method with MEGA software version 11 and 1000 bootstrap replicates were computed. Based on the classification of *A. thaliana* Dof and PbDof proteins were classified into subfamilies. Finally, using the online iTOL (https://itol.embl.de/, accessed on 8 July 2024) to polish the phylogenetic tree.

### 4.4. Analysis the Cis-Acting Elements of PbDof Genes

TBtools-IIv2.10 was used to obtain the sequence of 2000-bp upstream from each *PbDof* genes. PlantCARE (https://bioinformatics.psb.ugent.be/webtools/plantcare/html/, accessed on 5 July 2024) was used to obtain the *cis*-acting elements in the promoter region of the *PbDof* and visualize using the ‘Simple Bio Sequence Viewer’ function in TBtools-IIv2.10.

### 4.5. Analysis of SSR Loci

The MISA (https://webblast.ipk-gatersleben.de/misa/, accessed on 11 July 2024) web online tool was employed to analyze the SSR loci in the *PbDof* genes.

### 4.6. Gene Structure Analysis and Conserved Motif Recognition

Employing TBtools and relying on the genome annotation information of *P. bournei*, analyze the intron and exon characteristics of the *PbDof* gene. The PbDof protein sequence was imported into the MEME website (https://meme-suite.org/meme/, accessed on 4 July 2024), and the number of motifs was set to 10 for analysis and prediction. The Batch-CD-search function in the website NCBI (https://www.ncbi.nlm.nih.gov/Structure/bwrpsb/bwrpsb.cgi, accessed on 4 July 2024) was employed to analyze the domain contained in the amino acid sequence of *PbDof*. Finally, TBtools-IIv2.10 software was used to visualize the results of these three analyses more intuitively.

### 4.7. Abiotic Stress Experiment

The experimental materials are one-year-old *P. bournei* seedlings provided by Fujian Academy of Forestry. These materials have been subjected to drought, heat and strong light stress, respectively. For each stress treatment, three biological replicates have been set up. In each treatment, the materials are divided into a control group and a treatment group, with each group containing 10 individuals.

In the drought stress experiment, seedlings with consistent growth vigor are soaked in a 1/4 concentration of Hoagland nutrient solution for 10 days for root recovery treatment. Subsequently, the control group and the experimental group are cultured by adding PEG solutions with concentrations of 0% and 10%, respectively. In the high temperature stress experiment, *P. bournei* seedlings are placed in an artificial climate chamber with a temperature of 25 °C and a photoperiod of 12 h and cultured for 5 days. Then, the seedlings of the experimental group are placed in a culture box at 40 °C for stress treatment, while the control group is maintained at 25 °C. In the light stress experiment, the seedlings of the experimental group are placed under a light intensity of 20,000 μmol·m^−2^·s^−1^, and the control group is at 12,000 μmol·m^−2^·s^−1^. The temperature in the incubator is set at 25 °C. The photoperiod is set to 12 h of illumination.

For high temperature and drought experiments, samples are collected at 0 h, 4 h, 8 h, 12 h and 24 h, respectively. For light treatment, samples are collected at 0 h, 24 h, 48 h and 72 h, respectively. The collected samples are put into 2 mL EP tubes with steel balls and quickly frozen with liquid nitrogen to prevent RNA degradation and then stored in a refrigerator at −80 °C for future use.

### 4.8. RNA Extraction

For RNA extraction, the HiPure Plant RNA Mini Kit, (Shanghai, China) was employed. The obtained RNA was divided into aliquots and stored in a refrigerator at −80 degrees Celsius. Following the directions of the EasyScript^®^ One-Step gDNA Removal (Beijing, China) and cDNA Synthesis SuperMix kit (Beijing, China), reverse transcription was carried out using a PCR instrument. Subsequently, the cDNA obtained through reverse transcription was stored in a refrigerator at −20 °C By utilizing the Primer Premier v6.0 software, specific primers were designed based on the *PbDof* gene sequence that contains the most elements related to stress response, and qRT-PCR analysis was conducted. The RT-qPCR reaction conditions are as follows: pre-denaturation at 95 °C for 30 s; denaturation at 95 °C for 5 s, annealing at 60 °C for 60 s and extension at 50 °C for 30 s, with 40 cycles in total. The relative expression level of the gene was calculated by the 2^−ΔΔCT^ method. Then, GraphPad Prism10.0 software (https://www.graphpad.com/, accessed on 4 July 2024) was used to analyze the significance of differences in gene expression levels.

## 5. Conclusions

In this study, an in-depth exploration of 34 *Dof* gene families in *P. bournei* was conducted. The *Dof* genes were divided into eight subfamilies. Through phylogenetic analysis, it was found that *PbDof11* exhibits high homology with *AT5G60850.1* and *PbDof07* shows high homology with *AT3G50410.1*. It is speculated that they might possess the same functions. The results of collinearity analysis of genes indicated that 34 *PbDof* genes formed 24 pairs of homologous gene pairs. Frequent gene tandem duplication events occur in *PbDofs*, suggesting that *PbDof* genes may have a strong response to abiotic stress. By means of cis-acting element analysis, it is hypothesized that *PbDof* genes play roles in light response, stress response, hormone response, and plant growth and development. Under drought, high temperature, and light treatments, the expression patterns of seven *PbDof* genes were analyzed. It is speculated that *PbDof08* and *PbDof16* are the main genes involved in light response. Additionally, the mechanism of action of *Dof* genes when plants are under abiotic stress was also discussed. The above research provides valuable insights into the stress resistance research of *P. bournei* and the functional research of *Dof* genes.

## Figures and Tables

**Table 1:** List of the physicochemical properties and subcellar localization prediction on the *Dof* genes in *P. bournei*.

Gene Name	Gene ID	Size/Amino Acid	MW/Da	AI	PI	Grand Average of Hydropathicity	Instability Index	Subcellular Location
*PbDof01*	OF13769	219	24,402.05	50.32	9.11	−0.89	61.55	nucleus
*PbDof02*	OF13781	211	23,244.66	46.21	8.63	−0.913	58.84	nucleus
*PbDof03*	OF22513	218	24,294.06	43.39	8.94	−0.887	54.46	nucleus
*PbDof04*	OF22511	218	24,355.13	44.72	8.94	−0.909	56.74	nucleus
*PbDof05*	OF15518	334	36,064.60	51.74	8.34	−0.756	59.11	nucleus
*PbDof06*	OF15583	313	33,559.20	58.88	9.15	−0.696	55.51	nucleus
*PbDof07*	OF15599	221	22,871.37	51.72	6.8	−0.377	64.63	nucleus
*PbDof08*	OF11767	481	52,567.12	57.8	6.09	−0.614	48.91	nucleus
*PbDof09*	OF28193	435	47,623.71	58.71	6.79	−0.493	49.55	nucleus
*PbDof10*	OF04102	185	20,219.60	62.65	5.91	−0.385	55.55	nucleus
*PbDof11*	OF06877	308	33,262.88	54.19	8.66	−0.651	56.5	nucleus
*PbDof12*	OF22070	409	43,882.99	50.17	5.28	−0.642	61.86	cytosol
*PbDof13*	OF12386	208	23,194.70	57.87	8.84	−0.881	46.39	nucleus
*PbDof14*	OF12611	261	28,719.87	50.04	9.14	−0.785	57.05	nucleus
*PbDof15*	OF25919	496	54,439.93	47.3	5.58	−0.919	54.41	nucleus
*PbDof16*	OF07010	512	55,934.94	51.37	5.97	−0.822	60.98	nucleus
*PbDof17*	OF14679	308	33,871.94	46.46	6.16	−0.601	54.12	nucleus
*PbDof18*	OF01960	281	29,880.43	58.16	9.03	−0.495	55.66	nucleus
*PbDof19*	OF00444	122	13,812.73	36.83	9.59	−0.928	38.36	mitochondrion
*PbDof20*	OF15794	231	25,228.26	54.8	8.92	−0.758	52.42	nucleus
*PbDof21*	OF11216	297	32,586.47	54.16	8.63	−0.499	66.06	nucleus
*PbDof22*	OF10788	213	23,434.10	59.24	8.48	−0.738	52.58	nucleus
*PbDof23*	OF09224	263	29,534.67	52.5	6.94	−0.71	55.93	nucleus
*PbDof24*	OF02108	309	33,579.55	41.27	6.38	−0.559	60.87	nucleus
*PbDof25*	OF05260	276	29,370.88	57.85	8.47	−0.533	51.99	nucleus
*PbDof26*	OF26889	284	30,962.36	59.2	8.85	−0.725	49.15	nucleus
*PbDof27*	OF13878	312	34,446.71	45.98	6.25	−0.609	53.08	nucleus
*PbDof28*	OF16211	1191	128,485.74	46.85	8.87	−0.559	67.41	nucleus
*PbDof29*	OF02767	318	34,095.85	59.45	9.28	−0.634	57.08	nucleus
*PbDof30*	OF20349	149	16,404.73	58.48	9.15	−0.474	81.14	nucleus
*PbDof31*	OF00560	194	21,511.03	63.37	8.88	−0.786	50.21	nucleus
*PbDof32*	OF00385	267	29,474.87	48.93	8.49	−0.774	52.55	nucleus
*PbDof33*	OF21619	511	56,100.71	56.43	6.16	−0.769	52.97	nucleus
*PbDof34*	OF09116	150	16,469.67	45.33	9.54	−0.603	48.73	chloroplast

**Table 2:** SSR search results in *P. bournei* transcriptome.

SSR Type	SSR Number	SSR Percentage Content/%	SSR	Number of Repetitions
Trinucleotide	14	77.8	GAA	7
Quadnucleotide	1	5.50	GGGT	3
Pentanucleotide	1	5.50	TATCG	4
Hexanucleotide	2	11.20	ATGAAG CTCCAT	3
Total	18	100		

## Data Availability

The original contributions presented in this study are included in this article/Supplementary Material. Further inquiries can be directed to the corresponding author/s.

## Supplementary Materials

The following supporting information can be downloaded at: https://www.mdpi.com/article/10.3390/ijms252011147/s1.
